# Supplementary material for: Spatial control of secretory vesicle targeting by the Ync13–Rga7–Rng10 complex during cytokinesis
Source: bioRxiv. 2025 May 14:2025.05.13.653810. Preprint. [Version 1] doi: 10.1101/2025.05.13.653810 (PMC12132233; doi:10.1101/2025.05.13.653810)
Supplement: Supplementary file 1 [file NIHPP2025.05.13.653810v1-supplement-1.pdf]

bioRxiv preprint doi: https://doi.org/10.1101/2025.05.13.653810; this version posted May 14, 2025. The copyright holder for this preprint (which was not certified by peer review) is the author/funder, who has granted bioRxiv a license to display the preprint in perpetuity. It is made available under aCC-BY 4.0 International license.

Supporting Information

Table S1. ***S. pombe*** **strains used in this study.**

Strain	Genotype	Figure/video/reference
JW6068	*ync13-mECitrine-kanMX6 rga7-mCFP-kanMX6 ade6-210 leu1-32 ura4-D18*	Fig 1A
JW6128	*rng10-mCherry-natMX6 ync13-mECitrine-kanMX6 ade6-210 leu1-32 ura4-D18*	Fig 1A
JW8946	*ync13-tdTomato-natMX6 rng10-mEGFP-kanMX6 tom20-GBP-hphMX6 ade6-M210 leu1-32 ura4-D18*	Fig 1B
JW9010	*rga7-mEGFP-kanMX6 ync13-tdTomato-natMX6 tom20-GBP-hphMX6 ade6-210 leu1-32 ura4-D18*	Fig 1B
JW9339	*rng10-mEGFP-kanMX6 trs120-tdTomato-natMX6 tom20-GBP-hphMX6 ade6-21X leu1-32 ura4-D18*	Fig 1C
JW9435	*rga7-mEGFP-kanMX6 trs120-tdTomato-natMX6 tom20-GBP-hphMX6 ade6-21X leu1-32 ura4-D18*	Fig 1C
JW9315	*rga7-mEGFP-kanMX6 tom20-GBP-hphMX6 bgs4Δ::ura4*$^{+}$ *Pbgs4*$^{+}$*::RFP-bgs4*$^{+}$*-leu1*$^{+}$ *ade6-M210 leu1-32 ura4-D18 his3-D1?*	Fig 1D
JW9345	*rng10-mEGFP-kanMX6 tom20-GBP-hphMX6 bgs4Δ::ura4*$^{+}$ *Pbgs4*$^{+}$*::RFP-bgs4*$^{+}$*-leu1*$^{+}$ *leu1-32 ura4-D18 his3-D1? ade6-M210?*	Fig 1D
JW9397	*ags1Δ 3'UTR*$_{ags1}^{+}$*::ags1*$^{+}$*-Cherry:leu1*$^{+}$*:ura4*$^{+}$ *rga7-mEGFP-kanMX6 tom20-GBP-hphMX6 ade6-M210 his3-D1? leu1-32 ura4-D18*	Fig 1E
JW9394	*ags1Δ 3'UTR*$_{ags1}^{+}$*::ags1*$^{+}$*-Cherry:leu1*$^{+}$*:ura4*$^{+}$ *rng10-mEGFP-kanMX6 tom20-GBP-hphMX6 ade6-M210 his3-D1? leu1-32 ura4-D18*	Fig 1E
JW10114	*tom20-GBP-hphMX6 smi1-tdTomato-kanMX6 rga7-mEGFP-kanMX6 ade6-210 leu1-32 ura4-D18*	Fig 1F
JW7782	*ync13-mECitrine-kanMX6 rga7-13Myc-hphMX6 ade6-210 leu1-32 ura4-D18*	Fig 2A
JW5730	*h*$^{+}$ *ync13-mECitrine-kanMX6 ade6-210 leu1-32 ura4-D18*	Fig 2A
JW6734	*h*$^{-}$ *rga7-13Myc-hphMX6 ade6-210 leu1-32 ura4-D18*	Fig 2A
JW9614	*rga7-13Myc-hphMX6 bgs4Δ::ura4*$^{+}$ *Pbgs4*$^{+}$*::GFP-bgs4*$^{+}$*-leu1*$^{+}$ *ade6-210? leu1-32 ura4-D18 his3-D1?*	Fig 2B
562	*h*$^{+}$ *bgs4Δ::ura4*$^{+}$ *Pbgs4*$^{+}$*::GFP-bgs4*$^{+}$*-leu1*$^{+}$ *leu1-32 ura4-D18 his3-D1*	Fig 2B; Cortes et al., 2005
JW6734	*h*$^{-}$ *rga7-13Myc-hphMX6 ade6-210 leu1-32 ura4-D18*	Fig 2B
JW10167	*h*$^{+}$ *rga7-13Myc-hphMX6 smi1-mEGFP-kanMX6 ade6-M210 leu1-32 ura4-D18*	Fig 2C
JW8905	*h*$^{-}$ *smi1-mEGFP-kanMX6 ade6-210 leu1-32 ura4-D18*	Fig 2C
JW6112	*h*$^{+}$ *rga7-13Myc-hphMX6 ade6-M210 leu1-32 ura4-D18*	Fig 2C
JW10194	*rga7-mEGFP-kanMX6 smi1-13Myc-hphMX6 ade6-M210 leu1-32 ura4-D18*	Fig 2C
JW10185	*h*$^{+}$ *smi1-13Myc-hphMX6 ade6-M210 leu1-32 ura4-D18*	Fig 2C
JW3660	*h*$^{-}$ *rga7-mEGFP-kanMX6 ade6-210 leu1-32 ura4-D18*	Fig 2C
JW5730	*h*$^{+}$ *ync13-mECitrine-kanMX6 ade6-210 leu1-32 ura4-D18*	Fig 3, A and B
JW8876	*rga7Δ::ura4*$^{+}$ *ync13-mECitrine-kanMX6 ade6-M21X leu1-32 ura4-D18*	Fig 3, A and B
JW8912	*rng10Δ::hphMX6 ync13-mECitrine-kanMX6 ade6-210 leu1-32 ura4-D18*	Fig 3, A and B
JW5969	*ync13-mECitrine-kanMX6 rlc1-mCherry-natMX6 ade6-210 leu1-32 ura4-D18*	Fig 3C
JW8895	*ync13-mECitrine-kanMX6 rlc1-mCherry-natMX6 rga7Δ::natMX6 ade6-M210 leu1-32 ura4-D18*	Fig 3C
JW8913	*ync13-mECitrine-kanMX6 rlc1-mCherry-natMX6 rng10Δ::hphMX6 ade6-210 leu1-32 ura4-D18*	Fig 3C

bioRxiv preprint doi: https://doi.org/10.1101/2025.05.13.653810; this version posted May 14, 2025. The copyright holder for this preprint (which was not certified by peer review) is the author/funder, who has granted bioRxiv a license to display the preprint in perpetuity. It is made available under aCC-BY 4.0 International license.

JW3660	*h$^-$ rga7-mEGFP-kanMX6 ade6 leu1-32 ura4-D18*	Fig 3D
JW6063	*h$^-$ ync13Δ::kanMX6 rlc1-tdTomato-natMX6 rga7-mEGFP-kanMX6 ade6 leu1-32 ura4-D18*	Fig 3D
JW9470	*kanMX6-3nmt1-ync13 rlc1-tdTomato-natMX6 rga7-mEGFP-kanMX6 ade6-M210 leu1-32 ura4-D18*	Fig 3D
JW3693	*rga7-mEGFP-kanMX6 rlc1-tdTomato-natMX6 ade6-M210 leu1-32 ura4-D18*	Fig 3, E and F
JW6063	*h$^-$ rga7-mEGFP-kanMX6 rlc1-tdTomato-natMX6 ync13Δ::kanMX6 ade6 leu1-32 ura4-D18*	Fig 3, E and F
JW5899	*rng10-mEGFP-kanMX6 rlc1-tdTomato-natMX6 ade6-M210 leu1-32 ura4-D18*	Fig 3G
JW9483	*rng10-mEGFP-kanMX6 rlc1-tdTomato-natMX6 ync13Δ::kanMX6 ade6 leu1-32 ura4-D18*	Fig 3G
JW6810	*rlc1-tdTomato-natMX6 ags1Δ 3'UTR$_{ags1}$$^+$::ags1$^+$-GFP:leu1$^+$:ura4$^+$ade6 leu1-32 ura4-D18*	Fig 4A
JW6808	*ync13Δ::kanMX6 rlc1-tdTomato-natMX6 ags1Δ 3'UTR$_{ags1}$$^+$::ags1$^+$-GFP:leu1$^+$:ura4$^+$ade6 leu1-32 ura4-D18*	Fig 4A
JW6152	*bgs4Δ::ura4$^+$ Pbgs4$^+$::GFP-bgs4$^+$-leu1$^+$ rlc1-tdTomato-natMX6 ync13Δ::kanMX6 leu1-32 ura4-D18 his3-D1? ade6?*	Fig 4, B and D
JW6153	*bgs4Δ::ura4$^+$ Pbgs4$^+$::GFP-bgs4$^+$-leu1$^+$ rlc1-tdTomato-natMX6 leu1-32 ura4-D18 his3-D1? ade6?*	Fig 4, B and D
JW5249	*GFP-bgs1-leu1$^+$ bgs1Δ::ura4$^+$ rlc1-tdTomato-natMX6 ade6-M210 leu1-32 ura4-D18*	Fig 4, C and E
JW6616	*GFP-bgs1-leu1$^+$ bgs1Δ::ura4$^+$ rlc1-tdTomato-natMX6 ync13Δ::kanMX6 ade6 ura4-D18*	Fig 4, C and E
562	*h$^+$ bgs4Δ::ura4$^+$ Pbgs4$^+$::GFP-bgs4$^+$-leu1$^+$ leu1-32 ura4-D18 his3-D1*	Fig 4F; Cortes et al., 2005
JW6752	*rng10Δ::kanMX6 bgs4Δ::ura4$^+$ Pbgs4$^+$::GFP-bgs4$^+$-leu1$^+$ ura4-D18 his3-D1*	Fig 4F
JW9061	*ync13-19-his5$^+$-kanMX6 rng10Δ::kanMX6 bgs4Δ::ura4$^+$ Pbgs4$^+$::GFP-bgs4$^+$-leu1$^+$ ade6? ura4 his3-D1? leu1-32?*	Fig 4F
JW9062	*ync13-19-his5$^+$-kanMX6 bgs4Δ::ura4$^+$ Pbgs4$^+$::GFP-bgs4$^+$-leu1$^+$ ade6? ura4 his3-D1? leu1-32?*	Fig 4F
JW6152	*ync13Δ::kanMX6 bgs4Δ::ura4$^+$ Pbgs4$^+$::GFP-bgs4$^+$-leu1$^+$ rlc1-tdTomato-natMX6 leu1-32 ura4-D18 his3-D1? ade6?*	Fig 4G
JW6153	*bgs4Δ::ura4$^+$ Pbgs4$^+$::GFP-bgs4$^+$-leu1$^+$ rlc1-tdTomato-natMX6 leu1-32 ura4-D18 his3-D1? ade6?*	Fig 4G
JW9428	*rng10Δ::hphMX6 rga7Δ::kanMX6 bgs4Δ::ura4$^+$ Pbgs4$^+$::GFP-bgs4$^+$-leu1$^+$ rlc1-tdTomato-natMX6 ura4-D18 leu1-32 his3-D1? ade6?*	Fig 4G
JW9432	*rng10Δ::hphMX6 bgs4Δ::ura4$^+$ Pbgs4$^+$::GFP-bgs4$^+$-leu1$^+$ rlc1-tdTomato-natMX6 ura4? leu1-32 his3-D1? ade6?*	Fig 4G
JW9430	*rga7Δ::kanMX6 bgs4Δ::ura4$^+$ Pbgs4$^+$::GFP-bgs4$^+$-leu1$^+$ rlc1-tdTomato-natMX6 ura4? leu1-32 his3-D1? ade6?*	Fig 4G
JW6731	*rlc1-tdTomato-natMX6 trs120-3GFP-kanMX6 ade6-M210 leu1-32 ura4-D18*	Fig 5, A-D; Fig S5
JW7318	*rlc1-tdTomato-natMX6 trs120-3GFP-kanMX6 ync13Δ::kanMX6 ade6 leu1-32 ura4-D18*	Fig 5, A-D; Fig S5
JW9461	*rlc1-tdTomato-natMX6 trs120-3GFP-kanMX6 rga7Δ::kanMX6 ade6-M210 leu1-32 ura4-D18*	Fig 5, A and B; Fig S5
JW9462	*rlc1-tdTomato-natMX6 trs120-3GFP-kanMX6 rng10Δ::hphMX6 ade6-M210 leu1-32 ura4-D18*	Fig 5, A-D; Fig S5
JW9459	*rlc1-tdTomato-natMX6 trs120-3GFP-kanMX6 rng10Δ::hphMX6 rga7Δ::kanMX6 ade6-M210 leu1-32 ura4-D18*	Fig 5, A and B; Fig S5

bioRxiv preprint doi: https://doi.org/10.1101/2025.05.13.653810; this version posted May 14, 2025. The copyright holder for this preprint (which was not certified by peer review) is the author/funder, who has granted bioRxiv a license to display the preprint in perpetuity. It is made available under aCC-BY 4.0 International license.

JW6153	*bgs4Δ::ura4*$^{+}$ *Pbgs4*$^{+}$*::GFP-bgs4*$^{+}$*-leu1*$^{+}$ *rlc1-tdTomato-natMX6 leu1-32 ura4-D18 his3-D1? ade6?*	Fig 6, A and C
JW8831	*ync13Δ::kanMX6 ade6 leu1-32 ura4-D18*	Fig 6, B and C
JW7638	*rga7-mCherry-natMX6 tom20-GBP-hphMX6 ade6-M210 leu1-32 ura4-D18*	Fig S1A
JW9005	*rng10-mCherry-natMX6 tom20-GBP-hphMX6 ade6* leu1-32 *ura4-D18*	Fig S1B
JW9465	*kanMX6-3nmt1-mECitrine-ync13 tom20-GBP-hphMX6 ade6-M210 leu1-32 ura4-D18*	Fig S1C
JW9490	*sec1-tdTomato-natMX6 tom20-GBP-hphMX6 ade6-M210 leu1-32 ura4-D18*	Fig S1D
JW6969	*tom20-GBP-hphMX6 bgs4Δ::ura4*$^{+}$ *Pbgs4*$^{+}$*::RFP-bgs4*$^{+}$*-leu1*$^{+}$ *ade6-M210 leu1-32 ura4-D18 his3-D1?*	Fig S1E
JW7608	*ags1Δ 3'UTR*$_{ags1}$$^{+}$*::ags1*$^{+}$*-Cherry:leu1*$^{+}$*:ura4*$^{+}$ *tom20-GBP-hphMX6 ade6-M210 leu1-32 ura4-D18*	Fig S1F
JW8868	*ync13-tdTomato-natMX6 tom20-GBP-hphMX6 ade6-210 leu1-32 ura4-D18*	Fig S1G
JW8947	*rng10-mEGFP-kanMX6 tom20-GBP-hphMX6 ade6-M210 leu1-32 ura4-D18*	Fig S1H
JW9314	*tom20-GBP-hphMX6 rga7-mEGFP-kanMX6 ade6-M210 leu1-32 ura4-D18 his3-D1?*	Fig S1I
JW9475	*kanMX6-3nmt1-mECitrine-ync13 rga7-mCherry–natMX6 tom20-GBP-hphMX6 ade6-M210 leu1-32 ura4-D18*	Fig S2A
JW9477	*kanMX6-3nmt1-mECitrine-ync13 rng10-mCherry-natMX6 tom20-GBP-hphMX6 ade6 leu1-32 ura4-D18*	Fig S2A
JW9488	*sec1-tdTomato-natMX6 kanMX6-3nmt1-mECitrine-ync13 tom20-GBP-hphMX6 ade6-M210 leu1-32 ura4-D18*	Fig S2B
JW9500	*sec3-mCherry-natMX6 kanMX6-3nmt1-mECitrine-ync13 tom20-GBP-hphMX6 ade6-M210 leu1-32 ura4-D18*	Fig S2C
JW9473	*kanMX6-3nmt1-mECitrine-ync13 ede1-mCherry-natMX6 tom20-GBP-hphMX6 ade6-M210 leu1-32 ura4-D18*	Fig S2D
JW9478	*kanMX6-3nmt1-mECitrine-ync13 fim1-mCherry-natMX6 tom20-GBP-hphMX6 ade6-M210 leu1-32 ura4-D18*	Fig S2E
JW9495	*clc1-mCherry-natMX6 kanMX6-3nmt1-mECitrine-ync13 tom20-GBP-hphMX6 his3? ade6-M21 ? leu1-32 ura4-D18*	Fig S2F
JW9450	*rng10-mEGFP-kanMX6 tom20-GBP-hphMX6 sec3-tdTomato-hphMX6 ade6-M210 leu1-32 ura4-D18*	Fig S2G
JW9498	*bgs4Δ::ura4*$^{+}$ *Pbgs4*$^{+}$*::RFP-bgs4*$^{+}$*-leu1*$^{+}$ *kanMX6-3nmt1-mECitrine-ync13 tom20-GBP-hphMX6 ade6-M210 leu1-32 ura4-D18*	Fig S2H
JW9471	*ags1Δ 3'UTR*$_{ags1}$$^{+}$*::ags1*$^{+}$*-Cherry:leu1*$^{+}$*:ura4*$^{+}$ *kanMX6-3nmt1-mECitrine-ync13 tom20-GBP-hphMX6 ade6-M210 leu1-32 ura4-D18*	Fig S2H
JW9436	*bgs1Δ::ura4*$^{+}$ *Pbgs1*$^{+}$*-tdTomato-bgs1*$^{+}$*:leu1*$^{+}$ *rga7-mEGFP-kanMX6 tom20-GBP-hphMX6 leu1-32 ura4-D18 his3-D1? ade6-M210?*	Fig S2I
JW9371	*bgs1Δ::ura4*$^{+}$ *Pbgs1*$^{+}$*-tdTomato-bgs1*$^{+}$*:leu1*$^{+}$ *rng10-mEGFP-kanMX6 tom20-GBP-hphMX6 leu1-32 ura4-D18 his3-D1? ade6 M210 ?*	Fig S2I
JW8890	*ync13-tdTomato-natMX6 tom20-GBP-hphMX6 rga7FBD-mEGFP-kanMX6 ade6 leu1-32 ura4-D18*	Fig S3B
JW8986	*ync13-tdTomato-natMX6 tom20-GBP-hphMX6 rga7::kanMX6 GFP-rga7(ΔF-BAR):leu1*$^{+}$ *ade6-M210 leu1-32 ura4-D18*	Fig S3C
JW8942	*ync13-tdTomato-natMX6 tom20-GBP-hphMX6 rng10(1-200)-mEGFP-kanMX6 ade6-M210 leu1-32 ura4-D18*	Fig S3D
JW8949	*ync13-tdTomato-natMX6 tom20-GBP-hphMX6 kanMX6-Prng10-mECitrine-rng10(201-1038) ade6-M210 leu1-32 ura4-D18*	Fig S3E
JW8952	*ync13-tdTomato-natMX6 tom20-GBP-hphMX6 rng10(1-750)-mEGFP-kanMX6 ade6-210 leu1-32 ura4-D18*	Fig S3F

bioRxiv preprint doi: https://doi.org/10.1101/2025.05.13.653810; this version posted May 14, 2025. The copyright holder for this preprint (which was not certified by peer review) is the author/funder, who has granted bioRxiv a license to display the preprint in perpetuity. It is made available under aCC-BY 4.0 International license.

JW8989	*ync13-tdTomato-natMX6 tom20-GBP-hphMX6 kanMX6-Prng10-mECitrine-rng10(751-1038) ade6-210 leu1-32 ura4-D18*	Fig S3G
JW6153	*bgs4Δ::ura4⁺ Pbgs4⁺::GFP-bgs4⁺-leu1⁺ rlc1-tdTomato-natMX6 leu1-32 ura4-D18 his3-D1? ade6?*	Fig S4, A and B
JW9566	*sec1-M2-his5⁺-kanMX6 bgs4Δ::ura4⁺ Pbgs4⁺::GFP-bgs4⁺-leu1⁺ rlc1-tdTomato-natMX6 leu1-32 ura4 his3? ade6?*	Fig S4A
JW9563	*bgs4Δ::ura4⁺ Pbgs4⁺::GFP-bgs4⁺-leu1⁺ rlc1-tdTomato-natMX6 trs120-ts1-his5⁺-kanMX6 his5? leu1-32 ura4 ade6?*	Fig S4B
JW3693	*rga7-mEGFP-kanMX6 rlc1-tdTomato-natMX6 ade6-M210 leu1-32 ura4-D18*	Fig S4C
JW9673	*cwg1-1 rga7-mEGFP-kanMX6 rlc1-tdTomato-natMX6 ade6-210? leu1-32? ura4-D18*	Fig S4C
JW5899	*rng10-mEGFP-kanMX6 rlc1-tdTomato-natMX6 ade6-M210 leu1-32 ura4-D18*	Fig S4D
JW9671	*cwg1-1 rng10-mEGFP-kanMX6 rlc1-tdTomato-natMX6 ade6-210? leu1-32 ura4-D18?*	Fig S4D
JW9672	*cwg1-2 rng10-mEGFP-kanMX6 rlc1-tdTomato-natMX6 ade6-M210 leu1-32 ura4*	Fig S4D
JW6731	*rlc1-tdTomato-natMX6 trs120-3GFP-kanMX6 ade6-M210 leu1-32 ura4-D18*	Video 1
JW7318	*rlc1-tdTomato-natMX6 trs120-3GFP-kanMX6 ync13Δ::kanMX6 ade6 leu1-32 ura4-D18*	Video 2
JW9462	*rlc1-tdTomato-natMX6 trs120-3GFP-kanMX6 rng10Δ::hphMX6 ade6-M210 leu1-32 ura4-D18*	Video 3

bioRxiv preprint doi: https://doi.org/10.1101/2025.05.13.653810; this version posted May 14, 2025. The copyright holder for this preprint (which was not certified by peer review) is the author/funder, who has granted bioRxiv a license to display the preprint in perpetuity. It is made available under aCC-BY 4.0 International license.

Figure S1. **Representative controls for the Tom20-GBP mistargeting experiments.** (A-I) Micrographs of DIC, 514/488/561 nm channels, and merged channels showing cells expressing Tom20-GBP and another indicated protein. Tom20-GBP does not bind to mCherry, RFP, or tdTomato so the tagged proteins cannot be recruited to mitochondria without proteins tagged with mEGFP or mECitrine. No signal bleed through between red (561 nm)/yellow (514 nm) or red (561 nm)/green (488 nm) channels. The brightness and contrast were adjusted the same as the experimental groups with three tagged proteins shown in Figs. 1 or S2 so some panels appear almost totally black. Bars, 5 µm.

Figure S2. **Positive and negative physical interactions revealed by mistargeting to mitochondria using Tom20-GBP.** Mislocalized Ync13 ectopically targets (examples marked with arrowheads) Rga7 and Rng10 **(A)**, Sec1 **(B)**, Bgs4 and Ags1 **(H)** to mitochondria, but cannot interact with the exocyst subunit Sec3 **(C)** or proteins Ede1 **(D)**, fimbrin Fim1 **(E),** and clathrin light chain Clc1 **(F)** in the endocytic pathway. Ync13 was overexpressed using the *3nmt1* promoter by growing exponentially in YE5S liquid medium for ~48 h before imaging. Rga7 and Rng10 cannot mistarget Sec3 **(G)** or Bgs1 **(I)** to mitochondria. Bars, 5 µm.

Figure S3. **Physical interactions between full length Ync13 and Rga7 or Rng10 truncations revealed by ectopic mistargeting to mitochondria by Tom20-GBP. (A)** Domain schematics of Rga7 and Rng10 (Liu et al., 2016; Liu et al., 2019). **(B-G)** Arrowheads mark examples of colocalization at mitochondria. Except Rng10-(1-200) in **(D)**, all other Rga7 and Rng10

bioRxiv preprint doi: https://doi.org/10.1101/2025.05.13.653810; this version posted May 14, 2025. The copyright holder for this preprint (which was not certified by peer review) is the author/funder, who has granted bioRxiv a license to display the preprint in perpetuity. It is made available under aCC-BY 4.0 International license.

truncations **(B, C, and E-G)** can mistargeting Ync13-tdToamto to mitochondria. Rga7FBD = Rga7(1-320) (Arasada and Pollard, 2015); Rga7(ΔF-BAR), Rga7 without the F-BAR domain. Bars, 5 µm.

Figure S4. **Localizations of Rga7, Rng10, and Bgs4 in temperature-sensitive mutants.** Cells grown exponentially at 25°C were shifted to 36°C for 4 h (A, C, D) or 2 h (B) before imaging. Rlc1-tdTomato as the ring marker. Bgs4 localization in *sec1-M2* **(A)** or *trs120-ts1* **(B)** mutant cells. Rga7 **(C)** and Rng10 **(D)** localization in *bgs4* mutants *cwg1-1* and *cwg1-2*. Bars, 5 µm.

Figure S5. **Trs120 accumulates at division site in *ync13Δ*, *rga7Δ*, *rng10Δ*, and *rga7Δ rng10Δ* cells.** Rlc1-tdTomato marks the position and diameter of the contractile ring. Trs120-3GFP accumulates inside the area with the ring in *ync13Δ* cells, but outside the ring area in *rga7Δ*, *rng10Δ*, and *rga7Δ rng10Δ* cells at division site. The middle focal planes along with the sum intensity projection of the GFP channel from a 2-min continuous movie (exposure time 200 ms) without delay are shown. Arrowheads mark Trs120-3GFP in cells with constricting ring. Cells were grown exponentially in EMM5S liquid media for ~48 h before imaging. Bars, 5 µm.

Movie legends

Videos 1-3. **Movement of Trs120-3GFP vesicles to the division site.** The movies track the movement of Trs120-3GFP vesicles from the cytoplasm to the division site in WT, *rng10*Δ, and *ync13*Δ cells using colored lines. The videos correspond to Fig. 5 and Fig. S5. Strains were grown exponentially in EMMS liquid media at 25°C for ~48 h before imaging in continuous movies without delay. Time, min : sec.

bioRxiv preprint doi: https://doi.org/10.1101/2025.05.13.653810; this version posted May 14, 2025. The copyright holder for this preprint (which was not certified by peer review) is the author/funder, who has granted bioRxiv a license to display the preprint in perpetuity. It is made available under aCC-BY 4.0 International license.

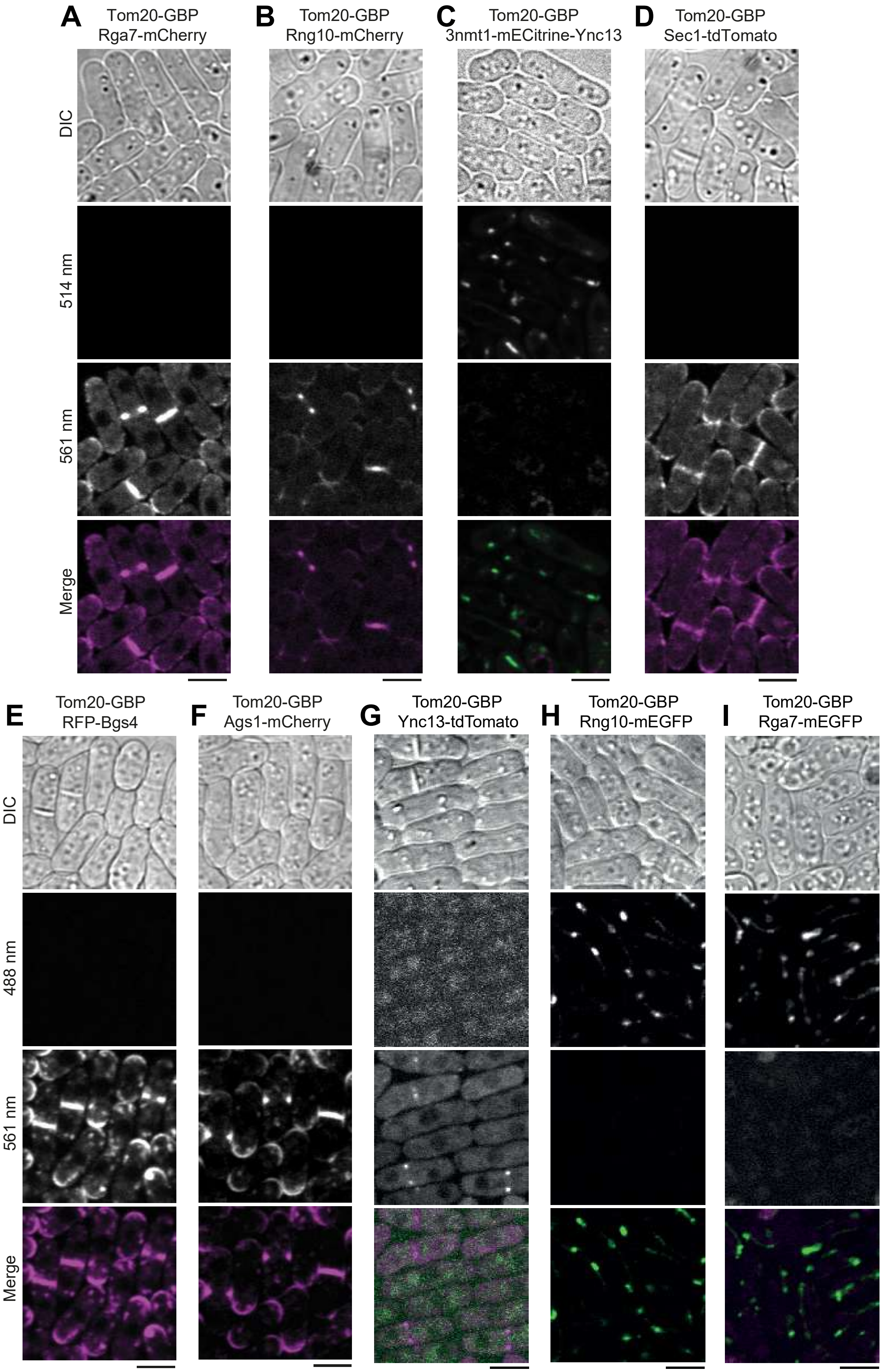

Figure S1

bioRxiv preprint doi: https://doi.org/10.1101/2025.05.13.653810; this version posted May 14, 2025. The copyright holder for this preprint (which was not certified by peer review) is the author/funder, who has granted bioRxiv a license to display the preprint in perpetuity. It is made available under aCC-BY 4.0 International license.

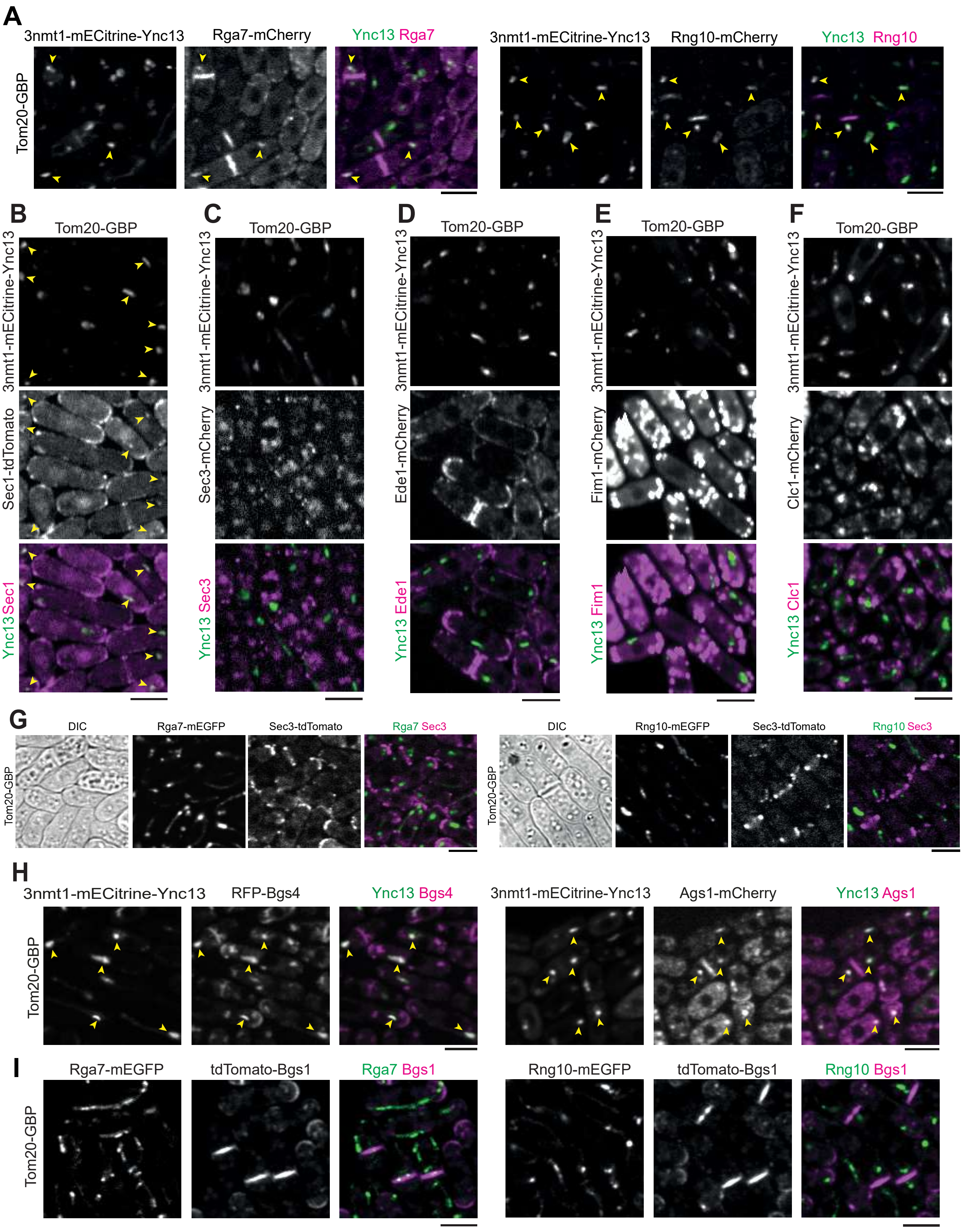

Figure S2

bioRxiv preprint doi: https://doi.org/10.1101/2025.05.13.653810; this version posted May 14, 2025. The copyright holder for this preprint (which was not certified by peer review) is the author/funder, who has granted bioRxiv a license to display the preprint in perpetuity. It is made available under aCC-BY 4.0 International license.

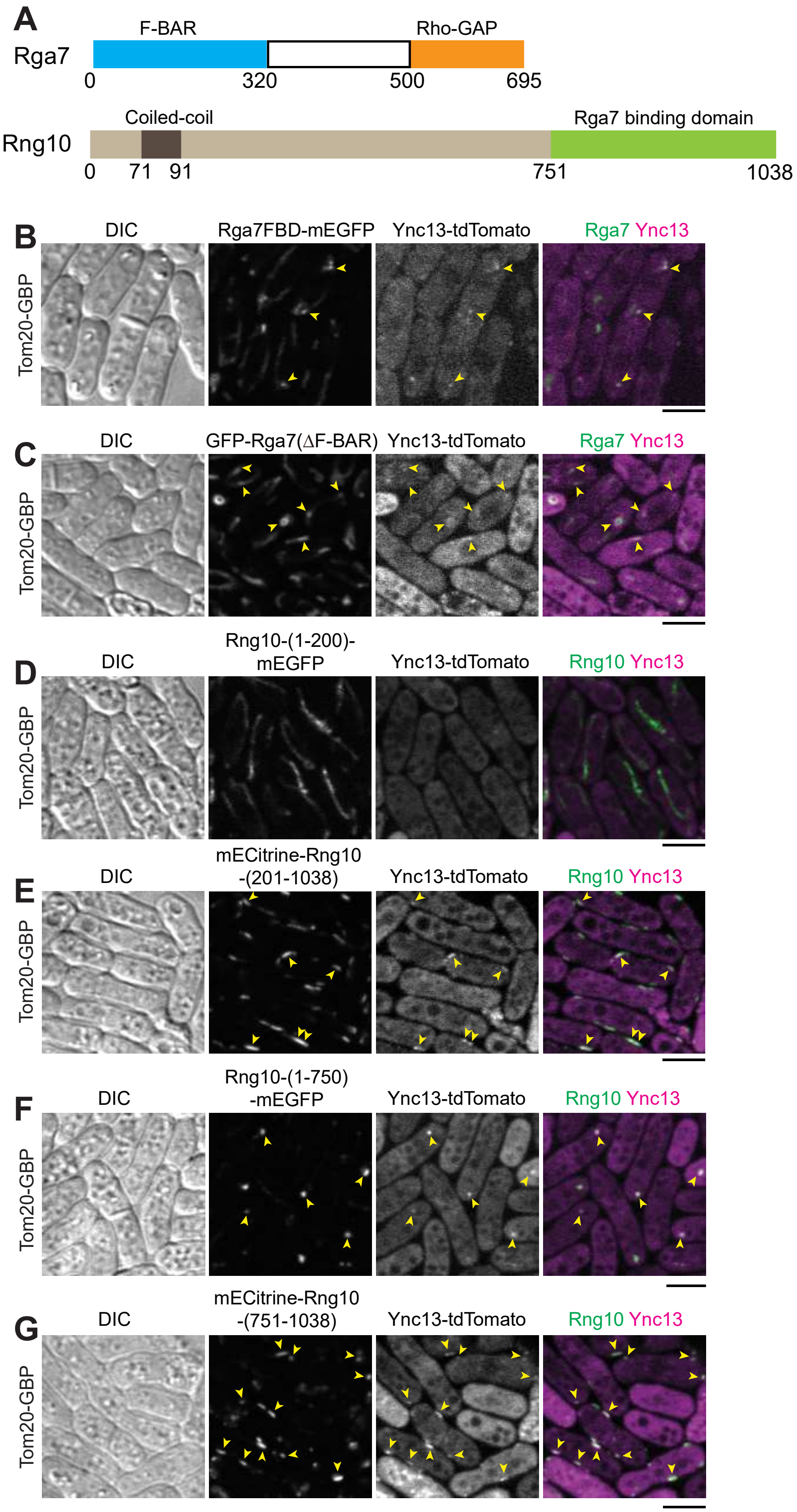

Figure S3

bioRxiv preprint doi: https://doi.org/10.1101/2025.05.13.653810; this version posted May 14, 2025. The copyright holder for this preprint (which was not certified by peer review) is the author/funder, who has granted bioRxiv a license to display the preprint in perpetuity. It is made available under aCC-BY 4.0 International license.

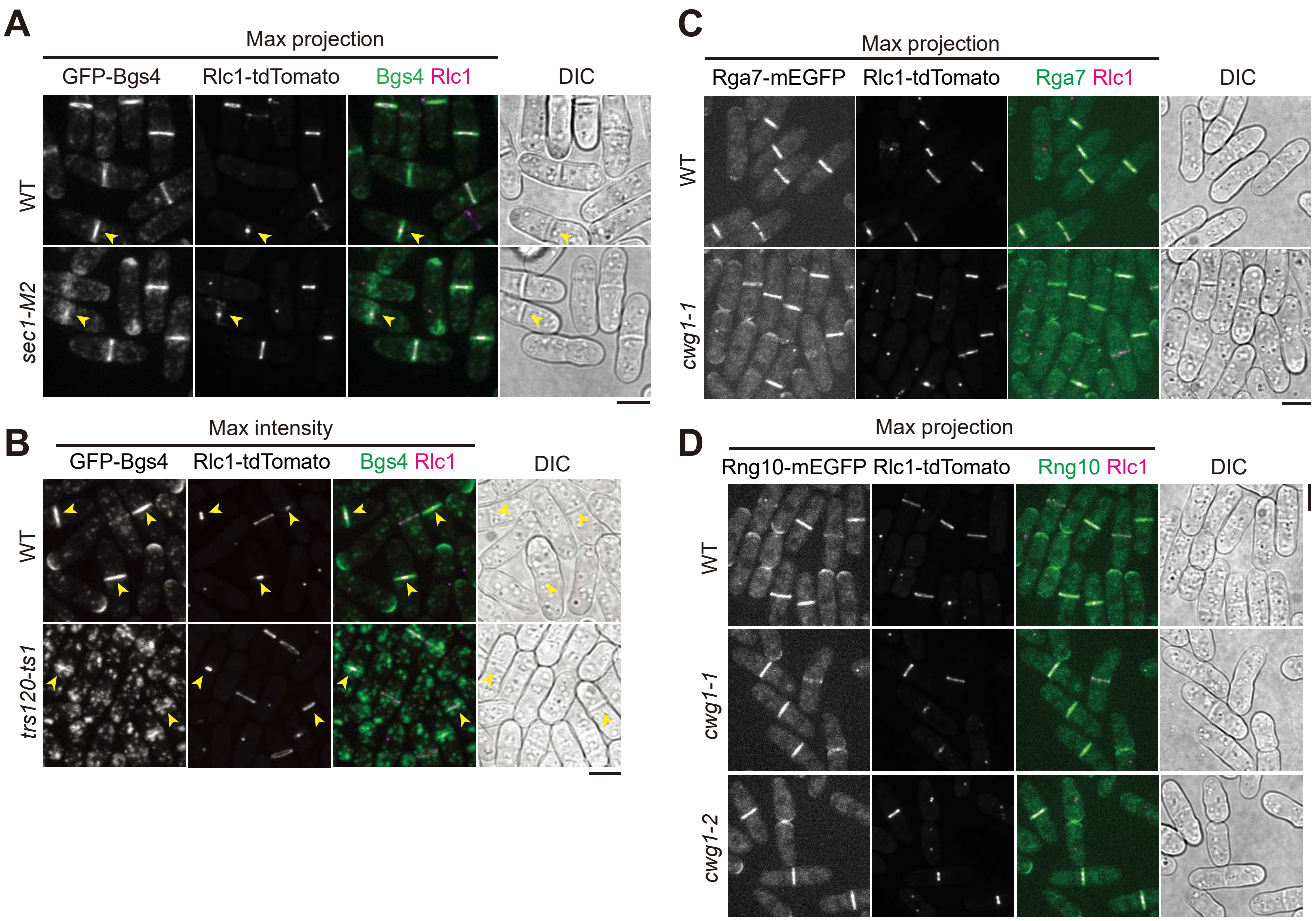

Figure S4

bioRxiv preprint doi: https://doi.org/10.1101/2025.05.13.653810; this version posted May 14, 2025. The copyright holder for this preprint (which was not certified by peer review) is the author/funder, who has granted bioRxiv a license to display the preprint in perpetuity. It is made available under aCC-BY 4.0 International license.

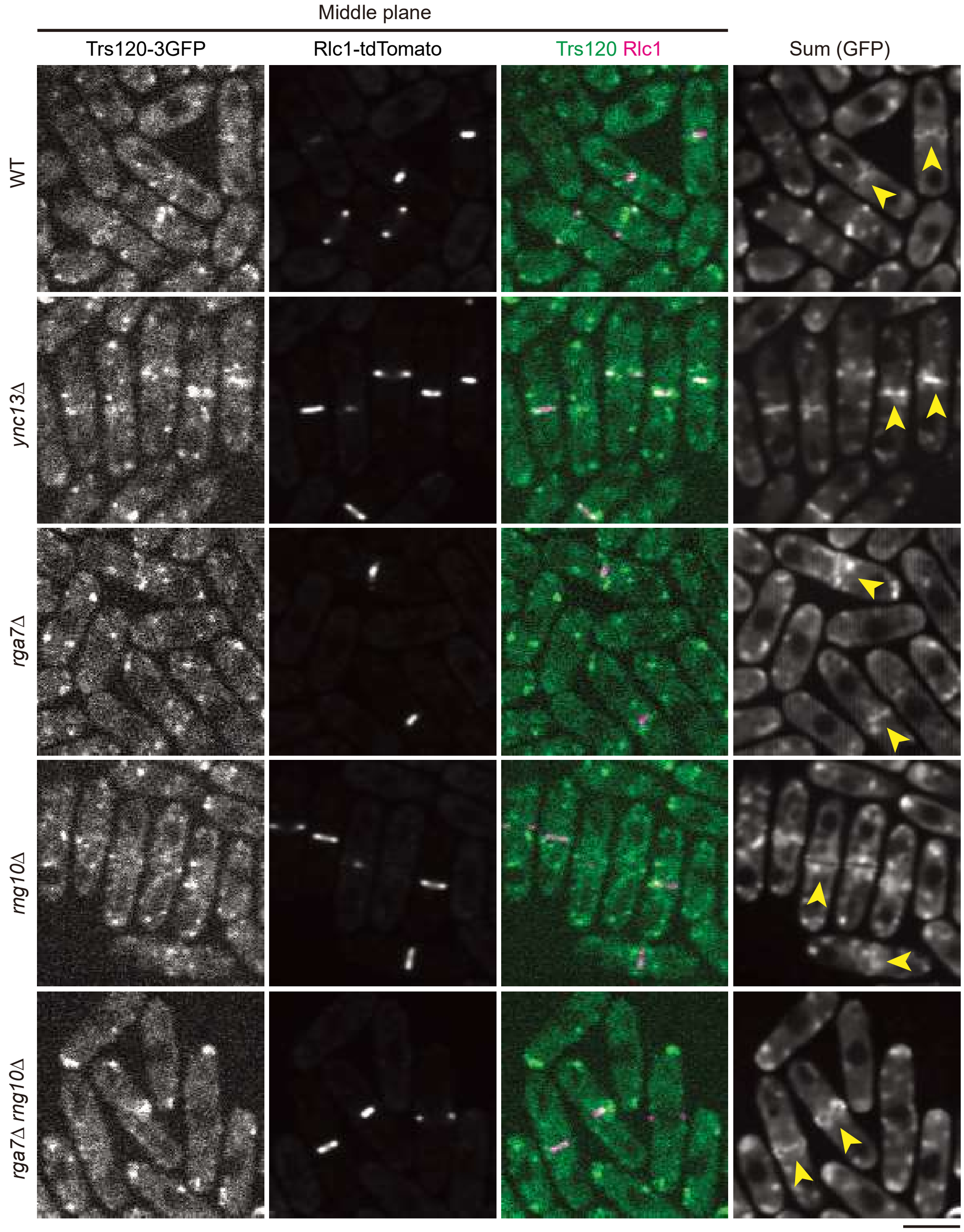

Figure S5
